# Stage IV Non-small Cell Lung Cancer Presenting as Supraventricular Tachycardia

**DOI:** 10.7759/cureus.4503

**Published:** 2019-04-20

**Authors:** Hina Amin, Debanik Chaudhuri

**Affiliations:** 1 Internal Medicine, State University of New York Upstate Medical University, Syracuse, USA; 2 Interventional Cardiology, State University of New York Upstate Medical University, Syracuse, USA

**Keywords:** supraventricular tachycardia, non-small cell lung cancer

## Abstract

Despite numerous advancements in diagnostics and treatment, lung cancer carries a high mortality rate. This is primarily attributable to the fact that the majority of patients present with stage III or IV disease and otherwise non-specific symptoms. In this article, we discuss a rare case of stage IV lung cancer presenting as supraventricular tachycardia secondary to cardiomediastinal involvement. Unfortunately, by the time the tumor had involved the mediastinum, surgical options were limited and treatment was largely palliative.

## Introduction

Lung cancer is a leading cause of cancer-related mortality in the United States (25.9% in 2017) [1]. The high mortality rate associated with lung cancer can be explained by aggressive course of this disease and frequent absence of any clinical manifestations until the tumor has progressed to an advanced stage. Non-specific symptoms such as cough, hemoptysis, dyspnea, and pleural effusions are thought to result from local and intra-thoracic tumor spread while distant spread can result in symptoms concerning the organ involved. Rarely, local tumor spread can result in involvement of mediastinal structures and manifest as the commonly known superior vena cava (SVC) syndrome, pericardial effusion, and even cardiac arrhythmias.

## Case presentation

**Figure 1:**
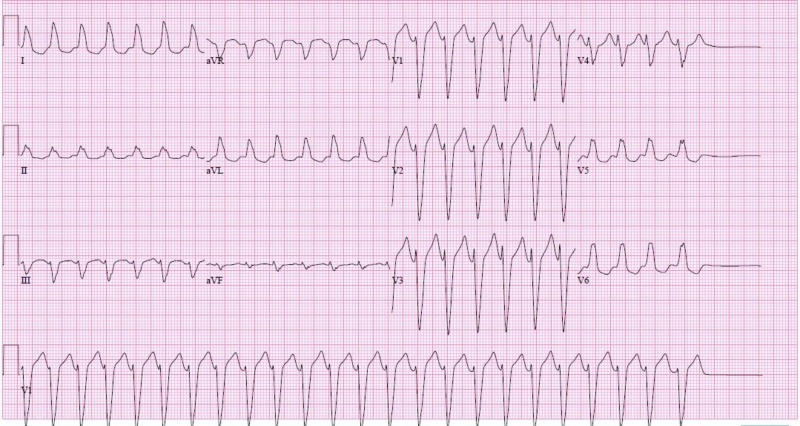
12-lead electrocardiogram (EKG) showing regular wide complex tachycardia with ventricular rate of 156 beats per minute

A 79-year-old male with a past history of aortic valve replacement presented to our hospital with sudden onset of lightheadedness and fatigue. On initial assessment, he was hypotensive with blood pressure (BP) 95/54, and pulse rate 168 beats per minute. Electrocardiogram (EKG) showed a wide complex tachycardia with a ventricular rate of 156 beats per minute (Figure 1).

**Figure 2:**
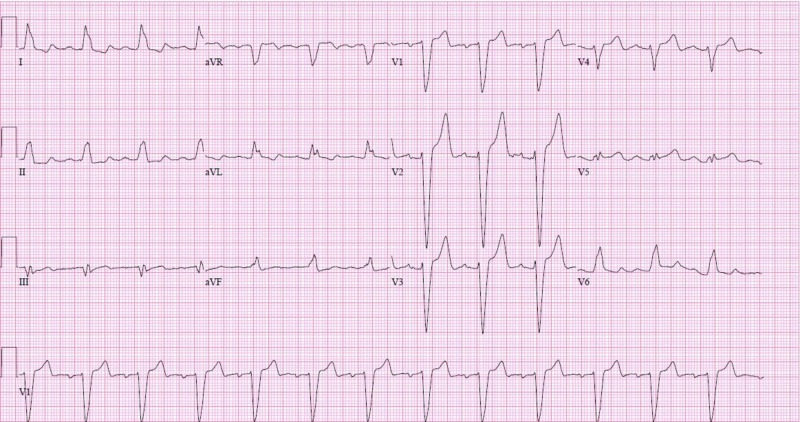
12-lead electrocardiogram (EKG) showing sinus rhythm with rate of 78 beats per minute, first-degree atrioventricular block, and left bundle branch block (LBBB)

Direct cardioversion was attempted followed by a loading dose of amiodarone and the patient subsequently electrically converted to sinus rhythm. Repeat EKG showed normal sinus rhythm with left bundle branch block (LBBB) and rate of 78/minute (Figure 2). 

**Figure 3:**
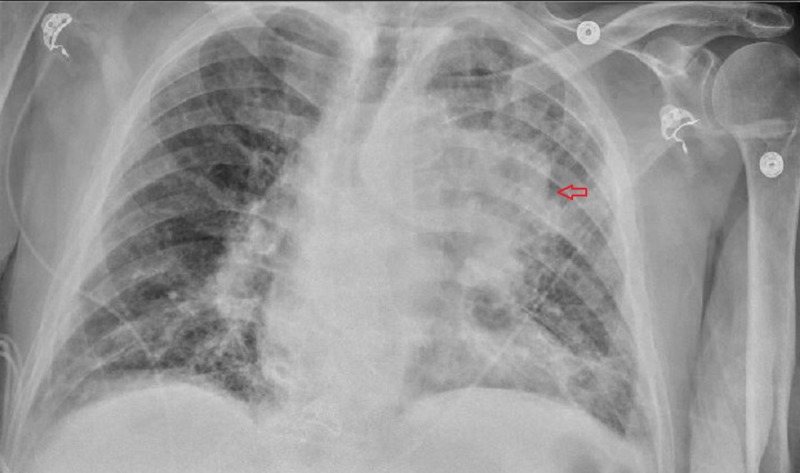
Chest X-ray showing left hilar and suprahilar opacity (arrow) with adjacent lung atelectasis

The patient was transitioned from amiodarone infusion to oral metoprolol. Further work-up revealed elevated creatinine, normocytic anemia, and elevated pro-brain natriuretic peptide (BNP). Echocardiogram showed a normal left ventricle ejection fraction, diastolic function, and a normally functioning bio-prosthetic valve. Chest X-ray showed a left hilar and suprahilar opacity (Figure 3).

**Figure 4:**
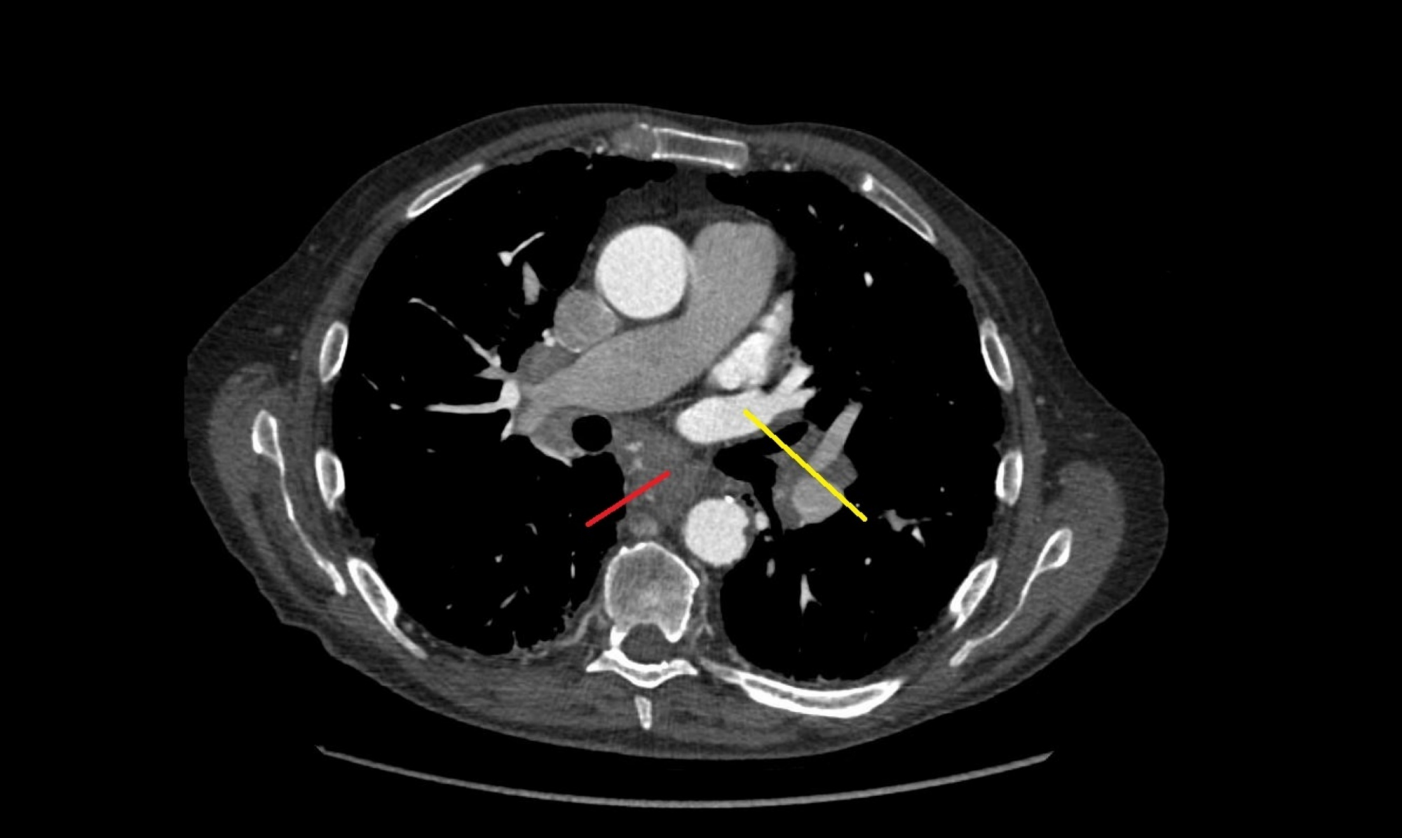
Computed tomography (CT) of the thorax with contrast showing left upper lobe mass with mediastinal involvement (red pointer) and encasement of left pulmonary vein (yellow pointer)

Computed tomography (CT) of the thorax with contrast further elaborated a 6.3 x 5.8 cm left upper lobe mass extending into the left hilum and encasing the left pulmonary arteries and veins (Figure 4).

Associated findings were mediastinal lymphadenopathy involving the left superior mediastinum and CT-guided biopsy of the lymph node showed metastatic lung adenocarcinoma. Given the extensive involvement of mediastinal structures, the tumor was deemed unresectable and a decision was made to treat it with palliative chemotherapy. Metoprolol was continued for rate control and the patient continued to remain in sinus rhythm.

## Discussion

Arrhythmias are an extremely rare complication of locally advanced lung cancer. Literature review of PubMed indexed cases has shown cases of mediastinal tumors leading to arrhythmias and only five of these were primary lung cancers. Cheng et al. reported a case of squamous cell carcinoma with invasion to the left atrium and left upper pulmonary vein leading to atrial fibrillation [2]. de Gregorio et al. described a case of squamous cell cancer infiltrating the right atrial wall and manifesting as chaotic atrial tachycardia and intermittent atrial fibrillation [3]. Peluso et al. noted a case of non-small cell lung cancer infiltrating the left ventricular apex and manifesting as atrial fibrillation [4]. Moreover at least two instances of ventricle myocardial metastases from primary lung cancer have been reported: Kinoshita et al. reported squamous cell lung cancer leading to ventricular tachycardia secondary to right ventricle myocardial metastases [5] while Ogino et al. described squamous cell cancer leading to new LBBB with myocardial metastases on autopsy [6]. Unlike benign non-infiltrating tumors which cause arrhythmia by mechanical stretching of myocardial fibers, invasive tumors can cause intramyocardial metastases which can alter the conduction pathways and trigger automaticity.

## Conclusions

The epidemic of lung cancer makes it important for physicians to be aware of its various atypical presentations. The purpose of this case was to highlight a rare mechanism of atrial arrhythmia and make readers aware of this unusual but consequential association. Unfortunately, by the time the tumor had involved the mediastinum, surgical options were limited. Screening guidelines have evolved to detect lung cancer in early stages thereby reducing the overall burden of advanced lung cancer.
